# What can hornworts teach us?

**DOI:** 10.3389/fpls.2023.1108027

**Published:** 2023-03-08

**Authors:** Eftychios Frangedakis, Alan O. Marron, Manuel Waller, Anna Neubauer, Sze Wai Tse, Yuling Yue, Stephanie Ruaud, Lucas Waser, Keiko Sakakibara, Péter Szövényi

**Affiliations:** ^1^ Department of Plant Sciences, University of Cambridge, Cambridge, United Kingdom; ^2^ Department of Systematic and Evolutionary Botany, University of Zurich, Zurich, Switzerland; ^3^ Zurich-Basel Plant Science Center, Zurich, Switzerland; ^4^ Department of Plant and Microbial Biology, University of Zürich, Zurich, Switzerland; ^5^ Department of Life Science, Rikkyo University, Tokyo, Japan

**Keywords:** terrestrialization of plants, land plants, polyplastidy, pyrenoid, RNA editing, evo-devo, plant-cyanobacteria symbiosis, plant-mycorrhizal symbiosis

## Abstract

The hornworts are a small group of land plants, consisting of only 11 families and approximately 220 species. Despite their small size as a group, their phylogenetic position and unique biology are of great importance. Hornworts, together with mosses and liverworts, form the monophyletic group of bryophytes that is sister to all other land plants (Tracheophytes). It is only recently that hornworts became amenable to experimental investigation with the establishment of *Anthoceros agrestis* as a model system. In this perspective, we summarize the recent advances in the development of *A. agrestis* as an experimental system and compare it with other plant model systems. We also discuss how *A. agrestis* can help to further research in comparative developmental studies across land plants and to solve key questions of plant biology associated with the colonization of the terrestrial environment. Finally, we explore the significance of *A. agrestis* in crop improvement and synthetic biology applications in general.

## 1 Introduction

As indicated by biochemical, morphological and molecular data, land plants evolved from aquatic green algae (Karol, 2001; Lewis and McCourt, 2004; Becker and Marin, 2009) around 500 million years ago. Land plants diverged into seven main groups: liverworts, mosses, hornworts, lycophytes, ferns, gymnosperms and angiosperms (Leebens-Mack et al., 2019). Our efforts to understand key events in the evolution of land plants are hindered by the fact that the majority of tractable land plant experimental systems belong to angiosperms (Szövényi et al., 2021). A few tractable non-seed plant models are available but only four are well-established: the fern *Ceratopteris richardii* (Plackett et al., 2015), the liverwort *Marchantia polymorpha* (Bowman et al., 2022), the moss *Physcomitrium paten*s (Rensing et al., 2020) and very recently the hornwort *Anthoceros agrestis* (Szövényi et al., 2015; Frangedakis et al., 2021b). Liverworts, mosses and hornworts form the monophyletic group of bryophytes, a deeply divergent lineage of plants. The recent development of a hornwort model, *A. agrestis*, makes comparative studies employing model systems from each of these deeply divergent clades feasible for the first time. This can eventually provide a more accurate insight into major events of land plant evolution (Dolan, 2009; Donoghue et al., 2021; Frangedakis et al., 2021a). Importantly, the development of a transformation technique for *A. agrestis*, and for three additional species (Waller et al., 2022), paves the way for detailed molecular and genetic studies to elucidate hornwort biology. Furthermore, it opens the way to experimentally study the enigmatic features of hornworts that are absent or rarely occur in other land plants, such as the single algal-like chloroplast per cell or the basal sporophyte meristem (Villarreal and Renzaglia, 2015; Frangedakis et al., 2021a). In this review we first provide an overview of the recently developed tools that enable experimental work on hornworts. We then highlight the key aspects of hornwort biology and their significance for general plant biology, applied plant science and synthetic biology. Finally, we discuss the challenges that need to be tackled in the future.

## 2 The significance of hornworts in understanding key questions of land plant evolution

Current phylogenetic evidence strongly supports the idea that land plants comprise two major monophyletic clades, the vascular plants (tracheophytes) and the bryophytes (Puttick et al., 2018; Leebens-Mack et al., 2019; Harris et al., 2020; Su et al., 2021; Harris et al., 2022). There is also accumulating evidence that within bryophytes, the hornworts are sister to the monophyletic clade of mosses and liverworts (Setaphytes) with a divergence time of approximately 400 MYA (Harrison and Morris, 2018; Puttick et al., 2018; Harris et al., 2022). This new phylogenetic backbone is in stark contrast to the traditional view that treated bryophytes as a paraphyletic grade of mosses, liverworts and hornworts (Buck et al., 2008). Importantly it has also revolutionized the way we think about character evolution in land plants. Traditional evolutionary hypotheses suggested that the common ancestor of land plants had a simple morphology, probably similar to extant bryophytes (Buck et al., 2008; Gerrienne et al., 2016). However, the discovery of bryophyte monophyly makes statements about the complexity and nature of the land plant common ancestor as well as character evolution in land plants challenging (Puttick et al., 2018; Harris et al., 2022). The monophyly of bryophytes and vascular plants implies that the land plant common ancestor could have had a haploid-dominant, a diploid-dominant, or an isomorphic (haploid and diploid phases with comparable complexity) life cycle. Therefore, the haploid-dominant life cycle and the organizational level of bryophytes may not be the ancestral state and could be the result of a reductive evolutionary process (Bowman et al., 2016; Chater et al., 2016; Harris et al., 2020; Harris et al., 2022). This is supported by recent findings indicating that the evolution of bryophytes might have been accompanied by massive gene losses after the split from the common ancestor of land plants (Clark et al., 2022; Harris et al., 2022). Furthermore, the new phylogenetic backbone of bryophytes combined with both genomic and evo-devo studies also imply that various complex traits could have been gained and lost among the three deeply divergent clades of bryophytes (Li et al., 2020; Rensing, 2020; Clark et al., 2022; Harris et al., 2022). Therefore, it is important to have at least one tractable model species for each major lineage of bryophytes. This will help to recognize and study the evolution of traits shared by most bryophyte groups and vascular plants, identify ancestral traits only retained by a specific group of bryophytes, as well as traits representing bryophyte-specific innovations. Such comparative analyses became increasingly possible with the establishment of the new hornwort model *A. agrestis.*

## 3 The hornwort model *Anthoceros agrest*is

**Figure 1:**
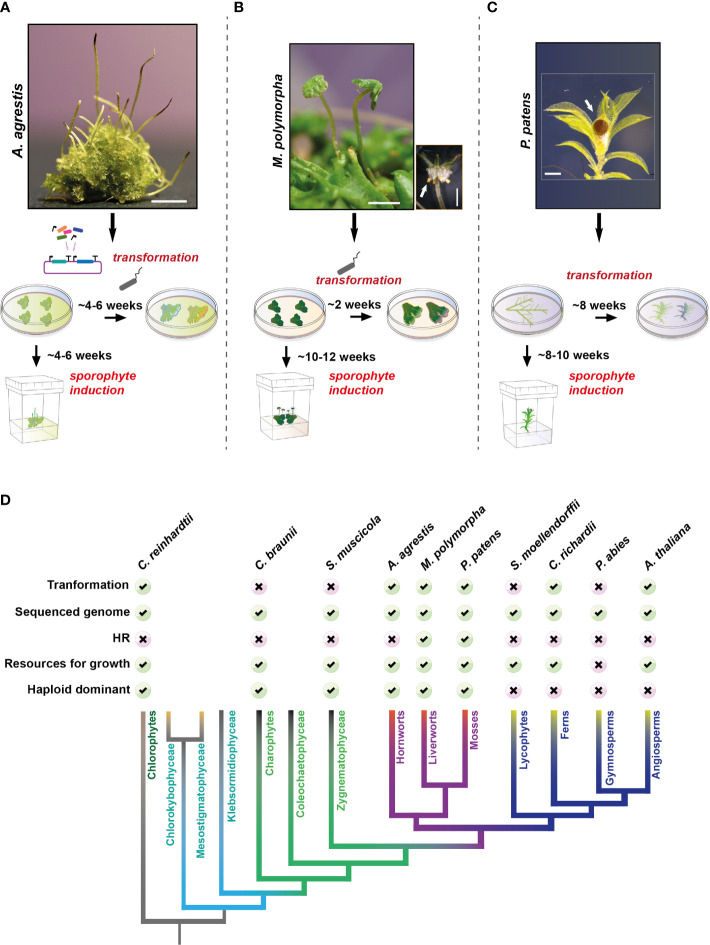
*Anthoceros agrestis* compared to other model plants. **(A–C)**: Top: Representative images of *Anthoceros agrestis* **(A)**, *Marchantia polymorpha* **(B)** and *Physcomitrium patens* **(C)** gametophytes and sporophytes. Bottom: Comparison of time required to obtain stable transgenic plants and sporophyte induction. Scale bars A: 2 mm, B: 2 mm, bottom right 1 mm and C: 0.5 mm. In **(B, C) **white arrow indicates sporophyte. **(D)** Phylogenetic relationships of the major lineages of land plants illustrating the monophyly of bryophytes and the monophyly of lycophytes, ferns, gymnosperms, and angiosperms (de Vries et al., 2016; Li et al., 2020). Comparison of model systems for key phylogenetic clades is shown on top.

For hornworts*, A. agrestis* (**Figure 1A**) has recently emerged as the model experimental system, with two geographic isolates (Oxford and Bonn) being available (Szövényi et al., 2015). Both *A. agrestis* isolates are derived from a single spore and can be routinely grown axenically. High quality genome assemblies are available (Li et al., 2020) and an efficient *Agrobacterium*-mediated transformation method, using regenerating thallus fragments, has been developed (Frangedakis et al., 2021b; Waller et al., 2022). Up to 100 and 40 stable transgenic lines can be obtained from 0.2 g of tissue for the Oxford and Bonn isolate, respectively. Protoplast isolation and transient transformation protocols are also available (Neubauer et al., 2022). Finally, a new growth medium for *A. agrestis* has been developed, that yields four times more tissue mass compared to the traditionally used KNOP medium (Gunadi et al., 2022). For cloning, the OpenPlant toolkit (Sauret-Güeto et al., 2020) originally developed for *M. polymorpha* has been adopted. The OpenPlant kit is a Golden Gate Cloning method, based on Type IIS restriction enzymes, that enables the fast generation of complex DNA circuits from standardized basic DNA parts (e.g., promoters, coding sequences and terminators).

In addition to the widely used CaMV 35S promoter, two native constitutive promoters have been developed for *A. agrestis*, the promoter regions of the *Elongation Factor 1 Alpha (Ef1a)* and the *Gamma Tonoplast Intrinsic Protein 1;1 (Tip1;1)* genes (Frangedakis et al., 2021b; Waller et al., 2022). Hygromycin is used as an antibiotic selection marker and chlorsulfuron as a herbicide selection marker. Four fluorescent proteins (eGFP, mVenus, mTurquoise2 and mScarlet) can be expressed successfully in *A. agrestis* without toxic effects. A set of targeting peptides for subcellular localization into the chloroplast, mitochondria, Golgi, Endoplasmic reticulum, peroxisomes, nucleus, cytoskeleton and the plasma membrane are also available (Waller et al., 2022). The palette of hornwort species that can be genetically modified was recently expanded by the addition of *Anthoceros punctatus*, which has been used as a model for plant symbiosis with cyanobacteria, *Leiosporoceros dussii*, which is the sister to all other hornworts and *Phaeoceros carolinianus* (Waller et al., 2022).

## 4 *A. agrestis* compared to other model plants

*A. agrestis* like any other bryophyte, has a gametophyte/haploid dominant life cycle (Buck et al., 2008). Axenic tissue propagation is simple and achieved by careful fragmentation of thallus tissue (Szövényi et al., 2015), however, *A. agrestis* does not tolerate high light intensities (Frangedakis et al., 2021b). The thallus tissue can be stored at 4° C for up to two months. Sporophyte induction is more efficient in the case of the Bonn isolate compared to the Oxford isolate and it takes about 6 weeks for mature sporophytes to develop (**Figure 1A**). Mature sporophytes can be removed and stored at 4° C for up to six months. In *A. agrestis* genetic studies are made easier, compared to angiosperms, by its small gene families, lack of redundancy and a dominant haploid phase in its life cycle (Szövényi, 2016; Li et al., 2020). For transformation, thallus fragments are co-cultivated with *Agrobacterium* and successful transformants are visible within 4-6 weeks after antibiotic/herbicide selection. Finally, *A. agrestis* provides an accessible platform for live-tissue microscopy where cell division can be easily tracked. CRISPR/cas9 genome editing technology is currently under development.

Model systems have been available for many years for the other two bryophyte groups, liverworts and mosses. For liverworts, *M. polymorpha* is a well-established model species (**Figure 1B**). *Agrobacterium* based transformation techniques are available and stable transformants can be obtained in approximately two weeks (Ishizaki et al., 2008; Kubota et al., 2013). The genome of *M. polymorpha* is available (Bowman et al., 2017; Diop et al., 2020; Montgomery et al., 2020) including extensive transcriptomic resources that are currently being improved (Kawamura et al., 2022). Homologous recombination is also feasible in *M. polymorpha*, however, it is not as efficient as in the moss *P. patens* (Ishizaki et al., 2013). It takes up to 12 weeks for mature sporophytes to develop. CRISPR/cas9 technology for genome editing is also well established (Sugano et al., 2014; Sugano et al., 2018). Transformation protocols are also available for two other liverworts: *Marchantia palacea*, commonly used for mycorrhizal symbiosis studies (Rich et al., 2021), and *Riccia fluitans* (Althoff and Zachgo, 2020).

For mosses, *P. patens* has been established as a model in 1924 by Wettstein (Wettstein, 1924) representing one of the oldest land plant model systems with the most extensive genomic resources among bryophytes (**Figure 1C**). Effective methods for *in vitro* propagation, protoplast-based, and *Agrobacterium*-mediated genetic transformations are available (Kammerer and Cove, 1996; Schaefer and Zrÿd, 1997; Cho et al., 1999; Li et al., 2010). Transgenic lines can be obtained in approximately 12 weeks. *P. patens* is unique for its ability to undergo homologous recombination with an efficiency similar to that of yeast (Schaefer and Zrÿd, 1997). It takes up to 10 weeks for mature sporophytes to develop, which is longer compared to *A. agrestis*. CRISPR/cas9 technology for genome editing is also available (Mallett et al., 2019; Guyon-Debast et al., 2021).

Other non-seed land plant model systems include lycophytes and ferns (**Figure 1D**). For lycophytes *Selaginella moellendorffii* has been proposed as a model and its genome was sequenced in 2011 (Banks et al., 2011). However, the lack of a transformation technique for S. *moellendorffii* and the length of time needed to complete its life cycle are major hurdles in genetic studies. For ferns, *C. richardii* was developed as a model system in 1960 (Hickok et al., 1987). *C. richardii* has a short life cycle under laboratory conditions, biolistic and *Agrobacterium* based transformation techniques (Plackett et al., 2014; Bui et al., 2015) are available and its genome was sequenced recently (Marchant et al., 2022). No CRISPR/cas9 protocol is available for editing *C. richardii*`s genome.

For angiosperms *Arabidopsis thaliana* is the system of choice (**Figure 1D**). *Agrobacterium* based transformation techniques are available and a high-quality reference genome is publicly available (http://www.arabidopsisbook.org/ and *Arabidopsis* Genome Initiative, 2000). Its life cycle can be completed within 6-8 weeks and an extensive set of molecular tools and genome editing techniques have been available for a long time. Several gymnosperm genomes are available (Rigault et al., 2011; Nystedt et al., 2013; Liu et al., 2021), however, their size and the length of their life cycles are major obstacles for laboratory-based experimentation.

Multiple model systems are available for green algae (**Figure 1D**). The most frequently used system is the unicellular green alga *Chlamydomonas reinhardtii* for which a genome assembly (Merchant et al., 2007) and efficient genetic transformation methods are available (Mussgnug, 2015). *Chara braunii* (Nishiyama et al., 2018) and *Spirogloea muscicola* (Cheng et al., 2019) have been proposed as multicellular green algae models, however despite their genomes being sequenced, genetic manipulation is still challenging.

Altogether, the hornwort model system performs well in comparison to other plant model (**Figure 1D**) systems in terms of ease of growth in laboratory conditions, life-cycle length and transformation efficiency. Nevertheless, important molecular tools, routinely used in the other two land plant models, are still under development.

## 5 The unique biology of hornworts

**Figure 2:**
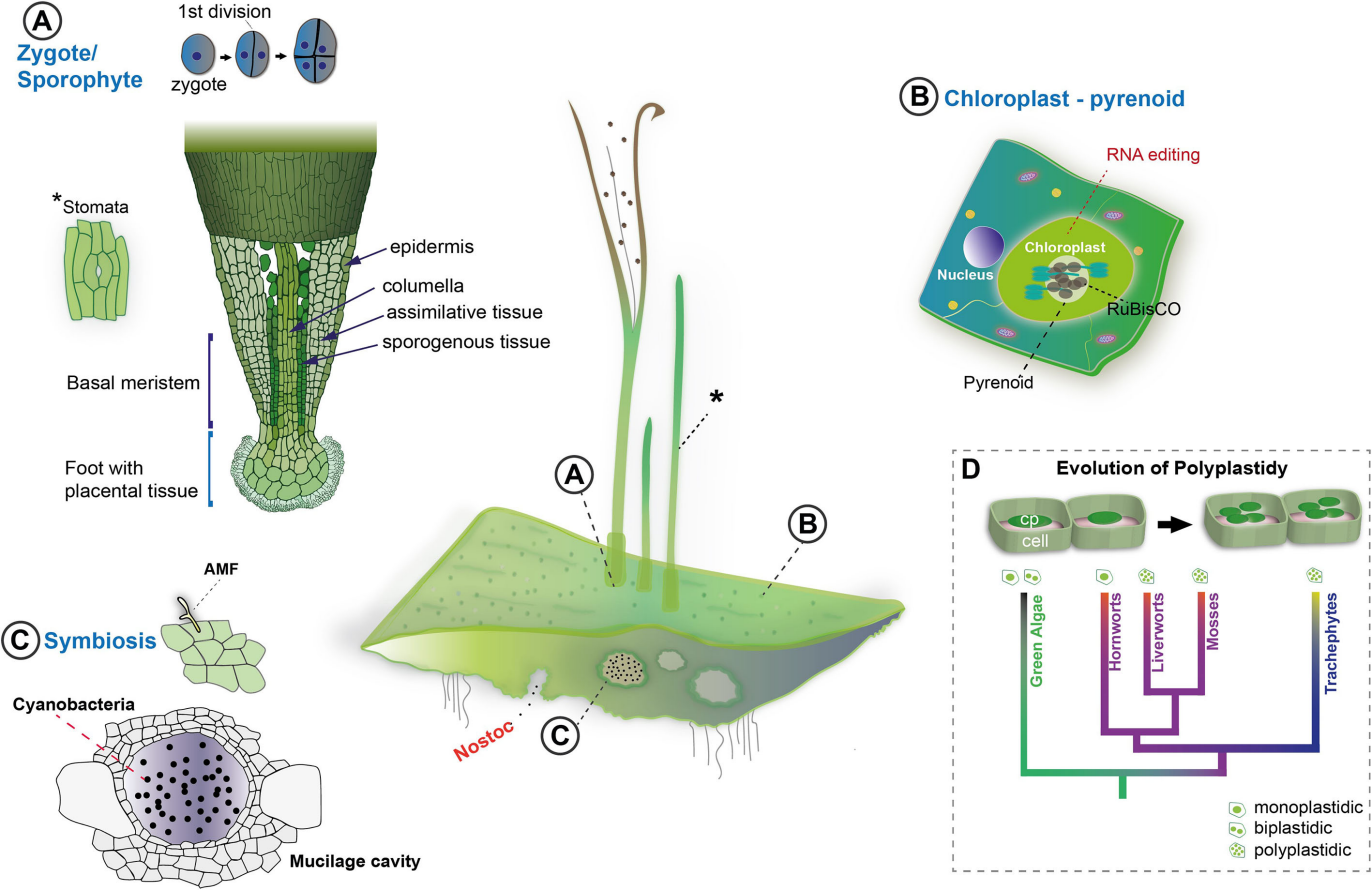
Unique biological traits of hornworts. Center: *Anthoceros agrestis* forms simple thallus tissues. Sporophytes grow on the gametophyte from thallus embedded zygotes, the first division of which is longitudinal. Stomata (indicated with "*") are present in the sporophyte **(A)**. Thallus cells have a single chloroplast that contains a pyrenoid and exhibits high levels of RNA editing **(B)**. The body of the thallus features mucilage cavities which play a role in cyanobacteria symbiosis **(C)**. Mucilage clefts are the entry point for *Nostoc* cyanobacteria. Arbuscular mycorrhizal fungi symbiosis can also be established in the thallus **(D)**. In general, *A, agrestis* thanks to its unique traits, can help to shed light on the evolution of key land plant innovations such as the evolution of polyplastidy (cp: chloroplast).

**Figure 3:**
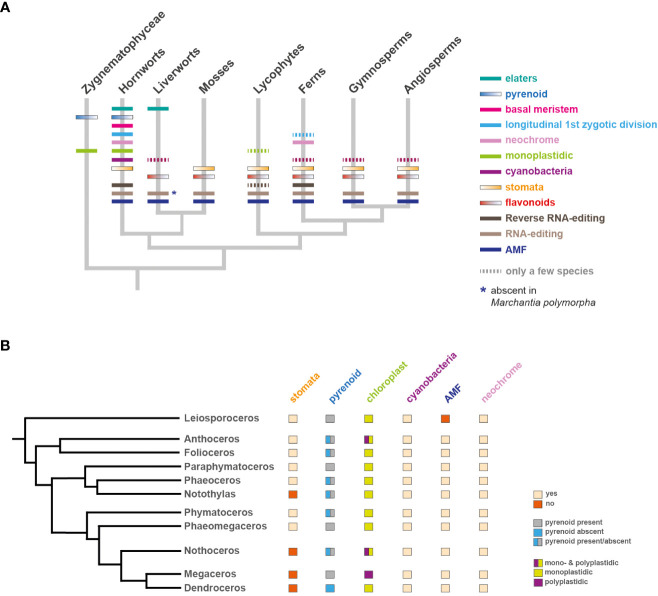
Occurrence of unique and evolutionary significant hornwort characters across land plants and within hornworts. **(A)** Land plant phylogeny (Li et al., 2020). The presence and/or absence of various traits is indicated with colored bars on corresponding tree branches. Zygnematophyceae are also biplastidic. **(B)** Phylogeny of hornworts based on (Villarreal and Renner, 2012). The presence and/or absence of various traits is indicated next to each genera name. Exception: stomata absent in *Folioceros incurvus*.

Hornworts have a handful of unique traits absent from other bryophytes or even any other land plants. The unique traits of hornworts include but are not restricted to (**Figures 2**, **3A**): (i) zygote and sporophyte development, (ii) a single chloroplast per cell, (iii) high-rates of RNA-editing and reverse editing, (iv) a pyrenoid based carbon concentrating mechanism, and (v) symbiotic relationships with arbuscular mycorrhiza fungi and cyanobacteria.

Hornworts comprise 11 families/genera which include: *Leiosporoceros, Anthoceros*, *Folioceros*, *Paraphymatoceros*, *Phaeoceros, Notothylas*, *Phymatoceros*, *Phaeomegaceros*, *Nothoceros*, *Megaceros* and *Dendroceros* (**Figure 3B**) (Villarreal and Renner, 2012). Interestingly, some of the unique hornwort characters vary even between different families. For example, the number of chloroplasts per cell or the presence of pyrenoids, stomata and arbuscular mycorrhizal symbiosis (Villarreal and Renner, 2012; Desiro et al., 2013; Frangedakis et al., 2021a; MacLeod et al., 2022) (**Figure 3B**). Thus, comparative studies within hornworts can help identify the genetic mechanisms that control these characters.

### 5.1 Zygote and sporophyte development

The orientation of the first zygote division in hornworts is longitudinal compared to the longitudinal axis of the archegonium unlike other land plants, with the exception of leptosporangiate ferns (Johnson et al., 2009) (**Figures 2A**, **3A**). The functional significance of this unique hornwort feature is unclear. It is hypothesized that the longitudinal division is a consequence of anatomical and mechanistic constraints (Shaw and Renzaglia, 2004). Unlike in mosses and liverworts where archegonia are superficial, hornwort archegonia and the zygotes are sunken in the gametophyte thallus and surrounded by gametophyte tissues. This mechanical constraint may have led to changing direction of the first division plane in hornworts and have likely independently evolved in leptosporangiate ferns (Johnson et al., 2009). Nevertheless, this hypothesis needs further testing and the genetic networks determining the first division plane must be thoroughly investigated. A potential component of such networks is the *A. agrestis* single *FLORICAULA/LEAFY (FLO/LFY)* gene, homologs of which have been shown to be necessary for the first zygotic division in *P. patens* (Maizel, 2005; Tanahashi et al., 2005).

**Figure 4:**
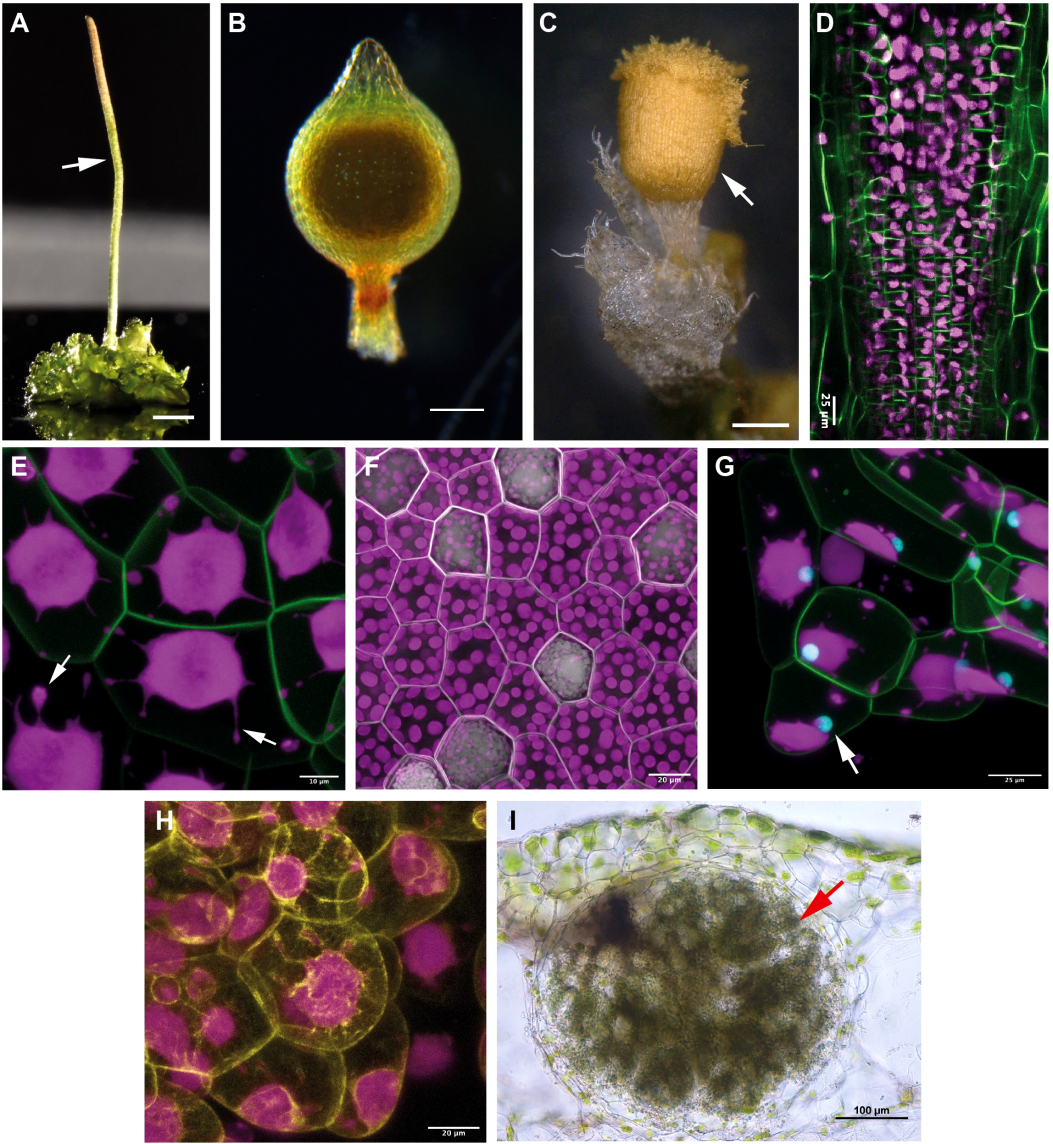
Sporophyte morphology, chloroplast and cellular features, and symbiotic interaction of the three bryophyte models. **(A)** *Anthoceros agrestis* Bonn gametophyte bearing a single sporophyte (indicated with white arrow). Scale bar 1.5 mm. **(B)** *Physcomitrium patens* sporophyte. Scale bar 200 μm. **(C)** *Marchantia polymorpha* sporophyte. Scale bar 500 μm. **(D)** Multiphoton microscopy images (whole mount) of the sporophyte base of *A*. *agrestis* Bonn expressing eGFP targeted to the plasma membrane. eGFP signal is visible in overlying cell rows of the basal meristem. Chloroplast autofluorescence in magenta. Scale bar: 25 μm. **(E)** Confocal microscopy image of *A*. *agrestis* Bonn gametophyte expressing eGFP targeted to the plasma membrane. Chloroplast autofluorescence in magenta. Stromules indicated with arrows. Scale bar: 25 μm. **(F)** Confocal microscopy image of *M. polymorpha* gemma with plasma membrane marked with mScarlet. Scale bar: 25 μm. **(G)** Confocal image of *A*. *agrestis* Oxford gametophyte tissue expressing eGFP targeted to the plasma membrane and mTurquoise2 targeted to the nucleus (indicated with arrow). Chloroplast autofluorescence in magenta. Scale bar: 50 μm. **(H)** Confocal microscopy image of *A*. *agrestis* Oxford with the actin labelled with mVenus. Scale bar: 20 μm. **(I)** Hand sections of *Anthoceros punctatus* thallus showing cavities colonized by cyanobacteria (indicated with arrow). Scale bar: 100 μm. Image provided by Masaki Shimamura.

Hornwort sporophytes are also unique amongst other bryophytes (**Figures 4A–C**) and land plants in general. A remarkable feature of the hornwort sporophyte is that most of it is formed by a multicellular basal meristem (Ligrone et al., 2012a) (**Figures 2A**, **3A**, **4D**). By contrast, the sporophyte of *P. patens* develops from an apical cell, a transient intercalary meristem, and various secondary meristems (Bower, 1935; Smith, 1955; Wardlaw, 1955; Kato and Akiyama, 2005; Lopez-Obando et al., 2022). Sporophyte tissues of the liverwort *M. polymorpha* do not contain a well-defined meristematic region (Ligrone et al., 2012a; Szövényi et al., 2019). The hornwort multicellular meristem is located at the base of the sporophyte, just above the foot connecting the sporophyte to the gametophyte, and continuously produces cells upwards that differentiate into the various cell types of the sporophyte (Bartlett, 1928; Ligrone et al., 2012a; Field et al., 2015). More specifically, the internal structure of the basal meristem resembles that of the root apical meristem of vascular plants (Ligrone et al., 2012a; Uchida and Torii, 2019; Dubrovsky and Vissenberg, 2021). Cells derived from the basal meristem are arranged in well-defined rows (**Figure 2A**, circles in 3D view) that give rise to the various tissue types of the sporophyte: the columella, the sporogenous tissue, the assimilative tissue and the epidermis. Each row consists of basally arranged meristematic cells that continuously differentiate into the major tissue types towards the apex of the sporophyte. Very little is known about the molecular mechanisms governing the development of this meristem and its evolutionary homology to the multicellular meristems of land plants is highly debated (Ligrone et al., 2012a; Ligrone et al., 2012b; Harrison and Morris, 2018; Fouracre and Harrison, 2022). Nevertheless, the continuous differentiation gradient present along the longitudinal axis of the sporophyte provides an excellent model to understand conserved features of cell fate determination across bryophytes and vascular plants, and potentially the origin of multicellular meristems in vascular plants.

The genetic network underlying the establishment and maintenance of the hornwort sporophyte meristem is poorly understood. *KNOTTED1-LIKE HOMEOBOX (KNOX1)* genes known to be critical for sporophyte development in *P. patens* (Sakakibara et al., 2008) and embryo formation in liverworts (Dierschke et al., 2021) are not present in the genome of *A. agrestis* (Li et al., 2020). While *KNOX1* genes are missing from the *A. agrestis* genome, a single *KNOX2* gene is present and preferentially expressed during sporophyte development. It can be speculated that the hornwort *KNOX2* gene may be able to compensate for the function of the absent *KNOX1* gene, however functional verification is needed to confirm such a hypothesis.

In angiosperms, a negative feedback pathway involving *CLAVATA* genes and the *WUSCHEL* transcription factor maintains the stem-cell population in the shoot apical meristem (SAM). In *A. thaliana* there are three *CLAVATA* genes, *CLV1*, *CLV2* and *CLV3*. *CLV3* encodes a small secreted protein and is expressed in the L1 and L2 layers of the SAM central zone while *CLV1* and *CLV2* genes encode receptor-like proteins and are expressed mainly in the L3 (Clark et al., 1997; Jeong et al., 1999). CLV3 interacts with CLV1 and CLV2 (Müller et al., 2008) to limit the *WUS* expression zone and consequently to limit the size of the stem cell/central zone (Reddy and Meyerowitz, 2005). Finally, *WUS* positively regulates *CLV3* activity, generating a feedback loop that stabilizes stem cell activity in the SAM. CLAVATA3/Embryo Surrounding Region-Related (CLE) peptides have been shown to promote the formation of gametophyte apical cells in *M. polymorpha* (Hirakawa et al., 2020; Hirakawa, 2022) and to antagonize stem cell formation in *P. patens* gametophyte (Cammarata et al., 2022). More recently, it has been shown that some CLE peptides (CLE40 in *Arabidopsis*) also have the capacity to promote meristematic activity in angiosperms (Schlegel et al., 2021), contrary to their canonical role antagonizing the meristem *via* WUSCHEL repression. Thus, experimental studies of CLE peptide/CLV receptors in hornworts will further clarify whether CLE peptides repressed or promoted meristematic activity in the land plant ancestor.

*YABBY* transcription factors have been shown to be involved in the regulation of lateral organ development, the proper maintenance of the SAM and initiation of axillary meristems in flowering plants (Bowman and Smyth, 1999; Siegfried et al., 1999; Kumaran et al., 2002; Goldshmidt et al., 2008; Tanaka et al., 2012). A *YABBY* gene has also been shown to be expressed in the simplex SAM, the microphyll primordia and the sporangium-primordia of the lycophyte *Huperzia selago* (Evkaikina et al., 2017). While no *YABBY* genes were found in *P. patens* or *M. polymorpha*, a single *YABBY* gene has been shown to be expressed in a sporophyte specific manner in the hornwort *A. agrestis* (Li et al., 2020). Investigating the functional role of the single hornwort *YABBY* gene will give insight into its potential role during basal meristem regulation and thus about the role of YABBY transcription factors during the evolution of land plant body plans in general.

The hornwort sporophyte also bears stomata (**Figures 2A**, **3A**). Stomata also occur in mosses but are absent in liverworts. (Renzaglia et al., 2017; Harrison and Morris, 2018; Clark et al., 2022). Unlike stomata of vascular plants, stomata of hornworts do not respond to environmental cues, they remain open once the stomata pore is formed and eventually collapse, facilitating sporophyte dehiscence and subsequent spore dispersal. In mosses stomata also play a role in sporophyte dehiscence but do respond to environmental cues such as light and abscisic acid (Chater et al., 2011; Lind et al., 2015). While the function and morphological features of moss stomata differ from those of vascular plants, their development is regulated by a set of orthologous genes (Pressel et al., 2014; Chater et al., 2017; Clark et al., 2022). The development, morphology and function of hornwort stomata further differs from those of mosses but shows remarkable similarity to Silurian-Devonian early land plant fossils (Edwards et al., 1998; Renzaglia et al., 2017). Elucidating the function of stomata in hornworts and determining the genetic network driving their development will help to resolve the long-standing debate about the ancestral function of stomata.

Studies of hornwort sporophytes can also help to understand the evolution and developmental biology of some unique features of bryophytes. For instance, both liverwort and hornwort sporophytes contain so-called elaters/pseudoelaters (elaters in liverworts and pseudoelaters in hornworts) aiding spore dispersal, while such structures are absent (likely lost) in all mosses (Ligrone et al., 2012a). Similarly, while most moss sporophytes contain a special opening structure (the annulus) enabling controlled dehiscence and regulated release of spores, most liverwort and all hornwort sporophytes open along two or more preformed slits (Crandall-Stotler and Stotler, 2008; Renzaglia et al., 2009). Additionally, while moss, liverwort and hornwort sporophytes are all nurtured by the gametophyte, nutrient transfer cells occur only in the gametophyte of hornworts while they are present in both the gametophyte and sporophyte tissues of several mosses and liverworts (Alfayate et al., 2000; Buck and Goffinet, 2000; Carafa et al., 2003; Crandall-Stotler and Stotler, 2008; Villarreal and Renzaglia, 2015). Finally, unlike mosses and liverworts, hornworts lack both water- and nutrient-conducting cells (Ligrone et al., 2000), with the evolutionary, physiological, and developmental aspects of this still not understood.

### 5.2 Chloroplast

#### 5.2.1 Monoplastidy-polyplastidy

Chloroplasts originated from an endosymbiotic event between a cyanobacterium and a eukaryotic cell over one billion years ago (Archibald, 2009). The ancient organism that resulted from the endosymbiotic event evolved into three main groups: 1) the Glaucophyta, 2) the Rhodophyta (red algae) and 3) the Viridiplantae (green algae and land plants), with various secondary endosymbiosis events giving rise to other algal groups such as stramenopiles and haptophytes (Keeling, 2004; de Vries and Gould, 2018). Gene transfers from the genome of the endosymbiotic cyanobacterium to the nuclear genome of the eukaryotic host saw the cyanobacterium progressively losing independence, thus evolving into a plastid (Bock and Timmis, 2008; de Vries et al., 2016; de Vries and Gould, 2018). However, this process remains incomplete: eukaryotic cells cannot produce a plastid *de novo* and only a plastid can make a new plastid by dividing *via* binary fission. Therefore, a daughter cell must inherit a plastid from its mother cell, otherwise the daughter cell lineage would be in an aplastidic state (de Vries and Gould, 2018). One solution is to have only one chloroplast per cell (monoplastidy) and to tightly link plastid division to cell division. Plastid division gene expression is coupled to cell cycle gene expression, with the mother cell only dividing once the chloroplast has multiplied so there is one chloroplast ready and available for each daughter cell. Most algal lineages are monoplastidic, including all the unicellular, basal branching groups in the chlorophyte green algae (de Vries and Gould, 2018). Some groups have escaped this bottleneck to evolve into a condition with multiple chloroplasts per cell: polyplastidy. This innovation appears to have evolved several times in photosynthetic eukaryotes, including within the streptophyte lineage that gave rise to the land plants (de Vries et al., 2016). Polyplastidy is suggested to be linked to the transfer of the plastid division *minicell* genes *MinD* and *MinE* from the chloroplast to the nuclear genome (Lopez-Juez and Pyke, 2005; de Vries and Gould, 2018). The increase in the number of plastids per cell coincided with the transition to terrestrial ecosystems and aided the development of macroscopic growth forms with specialized photosynthetic tissues. Multiple chloroplasts per cell allows for better photosynthetic efficiency and adaptation to variable environmental light conditions (Park et al., 1996; Königer et al., 2008; Ogasawara et al., 2013) in ways that are more difficult to achieve with the monoplastidic condition (Vaughn et al., 1992). Polyplastidic cells have a greater degree of back-up in case of damage or deleterious mutations in the chloroplast (Dutta et al., 2017; de Vries and Gould, 2018). The emergence of more complex body plans in vascular plants and seed plants also saw the evolution of new plastid types beyond the chloroplast (de Vries et al., 2016).

How polyplastidy evolved is poorly understood, and hornworts represent the ideal system to tackle this question. Most hornwort species, including the fully sequenced *Anthoceros* species, are monoplastidic (**Figures 2B**, **3B**, **4E**), however some groups (*e.g.*, *Megaceros*) are polyplastidic. Phylogenetic mapping suggests that bryophytes were originally polyplastidic, and within hornworts (**Figure 3B**) there were multiple independent transitions to monoplastidy (MacLeod et al., 2022). This provides opportunities to study the mono/polyplastidic transition both amongst the hornworts and within the bryophytes, with liverworts (for example the polyplastidic *M. polymorpha*, **Figure 4F**) and mosses providing convenient outgroups for comparison. By contrast such transitions are rare in other land plant groups (e.g. the giant plastids of the bizonoplast lycophytes, (Liu et al., 2020)). Furthermore, there are also cases of mono/polyplastidic transitions within one hornwort species, for example during *Megaceros* meristem regeneration. During the formation of an undifferentiated callus-like stage, the usual polyplastidic condition reverts to monoplastidy *via* an association between the chloroplast and nucleus that produces asymmetric cell divisions. After the emergence of apical cells and meristematic growth the usual polyplastidic state is restored (Burr, 1969). A similar transition from a polyplastidic to a monoplastidic conditions occurs in liverworts, such as *M. polymorpha.* Sporocytes (the cells that give rise to spores) have a single chloroplast per cells but during meiosis, they make a transition from a monoplastidic to a polyplastidic state (Renzaglia et al., 1994; Shimamura, 2016). Exploring the mechanism governing such transitions can provide insight into the evolution of polyplastidy.

In land plants, plastid division is largely independent from the cell cycle. However, the two processes cannot be entirely decoupled to avoid mis-segregation between daughter cells and the loss of plastids in one lineage. Birky’s model (Birky, 1983; Birky and Skavaril, 1984; Birky, 2001) suggests a way of controlling for this: if plastids occupy a sufficient percentage of cell volume (50%) or if there are a large enough number of plastids (>6) then the probability of inheritance is such that it is almost certain that each daughter cell will have at least one plastid, and from this can produce new additional plastids. Yet it is still unknown how land plant cells sense the number or size of the plastids they contain, or what the molecular mechanism linking plastid and cell division is. The process of plastid division by binary fission is carried out by the formation of contractile rings that are linked across the plastid inner and outer envelopes. The genes involved in the formation and function of these divisional rings are a combination of those inherited from the cyanobacterial ancestor (e.g. *MinD*, *MinE*) and innovations from the eukaryotic host (e.g. *Plastid Division (PDV)* genes) (Chen F. et al., 2018). Bryophytes also have a more streamlined repertoire of plastid-related genes versus model angiosperm species. This smaller gene repertoire, together with lineage-specific gene losses, provides a useful avenue to investigate their role in polyplastidy evolution. There have also been losses of plastid-related genes specifically within hornworts (MacLeod et al., 2022). Most notable in this regard are the distributions of *Accumulation and Replication of Chloroplasts 3* (*ARC3*) and the *Filamentous Temperature Sensitive Z (FtsZ)* gene family (*FtsZ 1, 2* and *3*) in hornworts. All green algae and land plants possess *FtsZ* genes. Within the bryophytes, mosses such as *P. patens* have multiple *FtsZ* genes (two *FtsZ1*, two *FtsZ2* and one *FtsZ3*), while liverworts (e.g. *M. polymorpha*) have one copy of each of *FtsZ1*, *FtsZ2* and *FtsZ3* (Grosche and Rensing, 2017). *ARC3* appeared early in chlorophyte evolution and is found throughout the land plants, with rare exceptions e.g. *P. patens* (though this may be compensated for by a *MinC*-like gene) (Miyagishima et al., 2011; Osteryoung and Pyke, 2014). Crucially, all hornworts analyzed thus far only possess a copy of *FtsZ1* and *FtsZ3*, that is, they lack *FtsZ2*, and have lost *ARC3* (MacLeod et al., 2022). *ftsZ2* and *arc3* mutants in *A. thaliana* and *P. patens* have impaired chloroplast division dynamics, displaying extreme phenotypes of large, misshapen chloroplasts (Martin et al., 2009; Schmitz et al., 2009; Zhang et al., 2013). The unique hornwort situation has been suggested to be a crucial factor in the evolution of monoplastidy, analogous to the *A. thaliana* and *P. patens ftsZ2* and *arc3* mutants. Another interesting observation is that the nucleus in an *A. agrestis* cell is always next to the plastid (**Figure 4G**). In addition, *A. agrestis* lines with the actin fluorescently labeled, reveal a close interaction of the actin-cytoskeleton with the chloroplast (**Figure 4H**). These observations point towards a potential mechanism that allows the coordination of plastid with cell division potentially *via* links with the cytoskeleton. Thus, hornworts provide a natural laboratory with simple, accessible systems to study FtsZ protein functions, FtsZ-ARC3 interactions, and the nucleus-plastid coordination.

Hornworts also provide an attractive system to study the least investigated *FtsZ* gene, *FtsZ3*, which is found in streptophyte green algae, bryophytes and lycophytes, but has been lost in ferns and seed plants (gymnosperms and angiosperms) (Grosche and Rensing, 2017) FtsZ3 differs from FtsZ1 or 2 in that the protein is localized to both the chloroplast and the cytoplasm in *P. patens*. *PpftsZ3* mutants possess misshapen chloroplasts and impaired phyllid growth, again distinct from phenotypes in *ftsz1* or *ftsz2* mutants (Martin et al., 2009). The fact that most traditional model plant species lack *FtsZ3* has hindered investigation into its function, yet the fact that it is ancestral in land plants makes it important in understanding the evolution of plastids and chloroplast division. One observation is that *FtsZ3* is correlated with a peptidoglycan layer between the chloroplast inner and outer envelopes, as is the case in cyanobacterial cell walls (and presumably in the ancestral endosymbiont). The suggestion is that FtsZ3 is somehow involved in forming or coordinating the peptidoglycan layer during plastid division (Grosche and Rensing, 2017), but the details surrounding this remain unclear (Chen C. et al., 2018). Hornworts provide an excellent opportunity to tackle this issue, having large, easily observable chloroplasts, established transgenic protocols and a single *FtsZ3* gene.

How polyplastidy evolved and controlled is a question of fundamental interest in plant biology, and the answers hold enormous biotechnological promise. Eventually controlling polyplastidy could facilitate applications such as the engineering of C4 traits in C3 crop species (e.g. to increase the chloroplast compliment in bundle sheath cells (Schuler et al., 2016; Stata et al., 2016)), manipulating plastid type interchange (Jarvis and López-Juez, 2013; Daniell et al., 2016; Solymosi et al., 2018) or producing morphologically-tailored plastids optimized for synthetic biology and protein biosynthesis (Daniell et al., 2016; Frangedakis et al., 2021c). Hornworts therefore represent a key system to study the regulatory mechanisms of chloroplast division, size and segregation, a “rosetta stone” for understanding the evolution of polyplastidy in land plants.

#### 5.2.2 Stromules

Hornworts allow other aspects of chloroplast morphology and function to be explored. One example of this are the numerous, prominent projections that extend from hornwort chloroplasts (see arrows, **Figure 4E**). These projections have been termed stromules (Frangedakis et al., 2021a), under the assumption that they are homologous and functionally equivalent to stromules in other plant groups (Gray et al., 2001; Natesan, 2005). Stromules are projections from plastids and can form independent of the cytoskeleton, in isolated chloroplasts (Brunkard et al., 2015). Despite a long history of observation (Gray et al., 2001), even in model angiosperm species such as *A. thaliana* and tomato, stromule biology remains mysterious (Waters et al., 2004; Schattat et al., 2012). There is experimental evidence for stromules having a role in chromoplasts during fruit ripening (Waters et al., 2004) and a role in reactive oxygen signaling during immune responses (Caplan et al., 2015), but other suggested functions in inter-plastid connectivity have been disproven (Schattat et al., 2012). In general, there is an inverse relationship between stromules and plastid density (Waters et al., 2004), including in chloroplast division mutants (Holzinger et al., 2008), and this is also the case for monoplastidic hornworts (Frangedakis et al., 2021a). This points towards stromules being involved in increasing chloroplast surface area, possibly for transfer of substances to/from the cytoplasm. This would be of greater importance for large hornwort chloroplasts, considering their high volume-surface area ratio. The *A. agrestis* genome has homologs of known stromule-related genes, such as the *Chloroplast Outer Envelope Protein 1* (Caplan et al., 2015), a prime target for further study.

### 5.3 RNA editing

RNA editing refers to the conversion of cytidine (C) to uridine (U) in plant mitochondria and chloroplasts (Maier et al., 1996; Gott and Emeson, 2000) and is found in most land plants including hornworts, but not in green algae (**Figure 3A**). C-to-U RNA editing is mediated by nuclear encoded proteins called Pentatricopeptide Repeat (PPR) proteins. Hornwort plastid genomes are unique, having one of the highest RNA editing rates amongst land plants executed by over 1400 PPR proteins (Gutmann et al., 2020). Furthermore, RNA editing rates appear to be variable within hornworts, providing a unique opportunity to investigate the relationship between PPR protein diversification and RNA editing rates (Kugita et al., 2003; Juan Carlos Villarreal et al., 2018; Gerke et al., 2020). Hornworts also exhibit a special type of RNA editing, called reverse editing (U-to-C), which is otherwise found only in some lycophytes and ferns (Knie et al., 2016; Knoop, 2022) (**Figure 3A**). Genetic studies in hornworts can provide valuable insight into the molecular basis and biological significance of this poorly understood phenomenon (Bernath-Levin et al., 2021).

Plant lineages capable of reverse editing, including hornworts, have evolved special types of PPR proteins (Gerke et al., 2020; Gutmann et al., 2020). Reverse RNA editing PPR proteins are of particular interest since they might have applications in chloroplast engineering similar to C-to-U RNA editing PPR proteins (Bernath-Levin et al., 2021; McDowell et al., 2022). For example, it has recently been reported that synthetic PPR proteins can be used to specifically direct C-to-U RNA editing in the chloroplast (Royan et al., 2021) Similarly, hornwort reverse RNA editing PPR proteins have also the potential to be engineered to direct reverse U-to-C editing, offering additional tools for the control of chloroplast transgenes.

### 5.4 Carbon concentrating mechanisms

To improve photosynthetic efficiency, various organisms have evolved mechanisms to increase intracellular carbon concentration. CO_2_ fixation is usually limited by the low CO_2_ attraction (Whitney et al., 2011) and catalytic rate (Bar-Even et al., 2011) of the Ribulose-1,5-bisphosphate carboxylase/oxygenase (RuBisCO) enzyme, and can be compensated by a mechanism, called carbon concentrating mechanism (CCM), that increases the CO_2_ concentration in the immediate vicinity of RuBisCO (Tcherkez et al., 2006). While this is achieved by employing complex multicellular structures in C4 and CAM plants, various algae and a single lineage of land pants, the hornworts, carry out biophysical carbon concentration at a single cell level in pyrenoids (**Figure 3A**) (Li et al., 2017). Pyrenoids are a chloroplast based biophysical CCM that incorporates transporters to actively move ${\text{HCO}}_{3}^{-}$ into the chloroplast, which is then converted into CO_2_ and concentrated around RuBisCO (Li et al., 2017; Meyer et al., 2017; Barrett et al., 2021).

To date, information on biophysical CCM and pyrenoid biology is mainly available for the unicellular alga *C. reinhardtii* and very little is known about hornwort pyrenoids (Barrett et al., 2021). Pyrenoids have repeatedly been gained and lost over the course of hornwort evolution (**Figure 3B**) enabling comparative analyses of pyrenoid-bearing and pyrenoid-absent species (Villarreal and Renner, 2012). This framework, combined with the currently established transformation techniques, provides an ideal opportunity to answer key questions related to pyrenoids in hornworts as well as to reveal conserved and divergent features with *C. reinhardtii* (Li et al., 2017; Frangedakis et al., 2021b; Waller et al., 2022). In particular:

(i) Measurements indicate that pyrenoid-bearing hornworts carry out active carbon concentration (Smith E. and Griffiths, 1996; Smith E. C. and Griffiths, 1996; Smith and Griffiths, 2000; Hanson et al., 2002; Meyer et al., 2008). Nevertheless, whether the CCM is inducible or rather constitutive is poorly known. Furthermore, the ultimate factors inducing CCM and their biological significance are unknown. Past and present atmospheric CO_2_ concentrations and habitat do not seem to correlate with the presence/absence of pyrenoids and CCM in hornworts (Villarreal and Renner, 2012). (ii) Pyrenoids are highly dynamic liquid phase separated structures in *C. reinhardtii.* By contrast, hornwort pyrenoids appear to be more stable and whether they form liquid phase separated bodies is unclear (Ligrone and Fioretto, 1987). (iii) Pyrenoids in *C. reinhardtii* consist mainly of RuBisCO scaffolded by a special protein matrix (Mackinder et al., 2016). While RuBisCO is concentrated in the pyrenoid in pyrenoid-bearing hornwort species and dispersed in the stroma in pyrenoid-absent species, the mechanism of pyrenoid assembly and components of the pyrenoid matrix are unknown (Vaughn et al., 1990; Vaughn et al., 1992). Whether pyrenoid assembly in hornworts occurs in an analogous way to *C. reinhardtii* is unclear (Barrett et al., 2021). (iii) Finally, the overall molecular mechanisms, the various enzymes/channels, and their localization within the hornwort cell and chloroplast are unknown and remain to be investigated.

Currently, there are attempts towards engineering algal pyrenoids into crops. This mainly includes proof of concept application in *A. thaliana* and tobacco (Adler et al., 2022). For example, engineering a chimeric *A. thaliana* RuBisCO by replacement of the two surface α-helices of its small subunit (S-subunit) with those of *C. reinhardtii*, results in a functional RuBisCO (Atkinson et al., 2017). *C. reinhardtii* RuBisCO S-subunit contains surface α-helices that have been shown to be necessary for the recruitment of RuBisCO into the pyrenoid *via* the interaction with the essential pyrenoid component 1 (EPYC1) (Engel et al., 2015; Mackinder et al., 2016). Recent evidence shows that co-expression of the chimeric *A. thaliana* RuBisCO with *C. reinhardtii* EPYC1 can result in functional pyrenoid-like structures in *A. thaliana* chloroplast (Atkinson et al., 2020). Given the vast evolutionary time separating chlorophytes from angiosperm crop species it can be hypothesized that it might be an easier endeavor to engineer a hornwort type pyrenoid into crops. Because the amino acid sequence of the hornwort RuBisCO S-subunit α-helices is more similar to that of *A. thaliana* compared to the *C. reinhardtii* one, introducing a hornwort EPYC1 analog to crops may induce pyrenoid formation with the native RuBisCO. Furthermore, pyrenoids produced this way may be more stable and have better biochemical properties than those induced using chimeric RuBisCO molecules and scaffolding proteins of algal origin.

### 5.5 Symbiotic interactions

Hornworts form beneficial (mutualistic) associations with arbuscular mycorrhizal (AM) fungi and cyanobacteria (Adams and Duggan, 2008; Rimington et al., 2020) (**Figures 2C**, **3B**). In exchange for photosynthetic carbon, the plant gains fixed nitrogen or phosphorus (Enderlin and Meeks, 1983; Desiro et al., 2013). These associations of plants with AM fungi and cyanobacteria played an important role in land plant evolution (Delaux and Schornack, 2021). Supplying the plants with essential nutrients might have enabled the adaptation to a terrestrial life in which organic matter was scarce (Rensing, 2018; Puginier et al., 2022).

#### 5.5.1 Arbuscular mycorrhizal fungi

Most hornwort species form a symbiosis with fungal partners (**Figure 3B**) (Desiro et al., 2013; Hoysted et al., 2017; Rimington et al., 2020). These associations involve two Mucoromycota subphyla, Mucoromycotina (that colonize 69% of hornworts) and Glomeromycotina (that colonize 78% of hornworts) (Rimington et al., 2020). Both the Glomeromycotina and Mucoromycotina are known to have played a key role in the plant adaptation to the land (Puginier et al., 2022). Often these different types of AM fungi occur simultaneously in the same thallus (Desiro et al., 2013). Fungal hyphae penetrate all parts of the thallus and occur inter- and intracellularly. Sometimes, the hyphae even interact with the cyanobacteria colonies (see next section) present in the mucilage cavities. While most hornwort species form a symbiosis with fungal partners, some species have apparently lost these symbioses (Read et al., 2000; Desiro et al., 2013). For example, the sister to all other hornworts *L. dussii*, has never been recorded in association with a fungus. In contrast, the family of Phymatocerotaceae has only been reported to occur in “a fungal association” (Rimington et al., 2020). Despite the presence of AM fungi in hornworts being well-documented using both morphological and molecular data (Schüßler, 2000; Desiro et al., 2013; Rimington et al., 2020), very little is known about the functional aspects of symbiosis and its underlying regulatory networks. The sequenced *Anthoceros* genomes contain the full complement (orthologs/homologs) of major common symbiosis pathway genes necessary to regulate the signaling between the host plant and the symbiont (Li et al., 2020). Nevertheless, the conservation of genetic networks governing the initiation, establishment and stabilization of symbiotic interaction has yet to be investigated. Evidence is mounting that the common ancestor of all land plants was capable of establishing symbiosis with AM fungi (Rich et al., 2021). Information about the gene network regulating AM fungal interactions in liverworts is emerging (Radhakrishnan et al., 2020; Rich et al., 2021; Kodama et al., 2022). However, due to the deep divergence between hornworts and liverworts some findings may represent liverwort-specific innovations and cannot be generalized. Therefore, establishing a hornwort system to study AM fungal interactions is of high significance. Revealing conserved regulatory mechanisms shared by hornworts and other land plants will help to reconstruct the ancestral symbiosis molecular tool kit. A hornwort study system will also enable studying the potential three-way interaction between plant host, cyanobacteria, and AM fungi. To do so, research in isolating AM fungi from various hornworts is in progress and initial experiments indicate that the symbiotic interaction can be reconstituted under axenic conditions (Ono et al., 1992).

#### 5.5.2 Cyanobacteria

Hornworts also establish a symbiosis with cyanobacteria providing the host plant with fixed nitrogen in exchange for photosynthates (**Figures 2C**, **3B**, **4I**) (Meeks and Elhai, 2002; Adams and Duggan, 2008). Unlike plant interaction with AM fungi, the endophytic interaction between plants and cyanobacteria is rarer and seems to have evolved independently in just a few phylogenetically diverse groups of land plants: in bryophytes (all hornworts and two species of liverworts), ferns (*Azolla*), gymnosperms (*Cycads*), and angiosperms (*Gunnera* and *Oryza*) (Meeks, 1998; Meeks and Elhai, 2002; Santi et al., 2013; Álvarez et al., 2020).

The cyanobionts hosted by hornworts are usually from the genus *Nostoc* spp. (Adams and Duggan, 2008) but can be phylogenetically diverse (Nelson et al., 2021; Rahmatpour et al., 2021). Hornworts host the cyanobacteria in mucilage cavities that can be accessed through ventral mucilage clefts, which superficially resemble stomata (Renzaglia et al., 2000; Villarreal A and Renzaglia, 2006). An exception is *L. dussii* that hosts cyanobacteria in canals that branch and form an integrated network within the thallus (de Vries and de Vries; Meeks, 2003).

Initiation of the cyanobacteria-plant symbiosis requires mobilization and chemical attraction of cyanobacteria. In the majority of plant-cyanobacteria symbioses this is achieved by molecules collectively called hormogonia-inducing factors (HIF) that are produced by the plant host and transform the cyanobacterial cells to hormogonia. Hormogonia are motile cells that can detach from the parent organism and function as dispersal units (Adams and Duggan, 2008; Liu and Rousk, 2022; Rousk, 2022). The hornwort-cyanobacteria symbiosis is relatively easy to reconstruct under axenic conditions, with *A. punctatus* being used as the model system to study the morphological, functional, molecular, and chemical processes underlying the symbiosis (Enderlin and Meeks, 1983). In *A. punctatus* (Enderlin and Meeks, 1983), a HIF is released when the hornwort is deprived of fixed nitrogen, enabling the colonization of the cyanobiont (Campbell and Meeks, 1989). Once the mucilage-rich cavities are colonized, the hornwort releases a hormogonia-repressing factor triggering the production of vegetative cyanobacterial filaments enriched with specialized N-fixing cells, the heterocysts (Campbell et al., 1997). While the cyanobacterial genes regulating symbiosis initiation, establishment, and stabilization are relatively well-investigated, very little is known about the host (de Vries and de Vries). It is thought that attraction of the cyanobiont may rely on some mechanisms conserved across the diverse lineages of land plants in which endophytic plant-cyanobacteria symbiosis occur (de Vries and de Vries). A recent RNA sequencing study identified a suite of candidate genes that might mediate the hornwort-cyanobacteria symbiotic relationship (Li et al., 2020; Chatterjee et al., 2022). These include a SWEET transporter, receptor kinases, and transcription factors but many questions remain to be answered.

Experimental tractability of the hornwort-cyanobacteria model system and the ability to genetically transform *A. punctatus* (Frangedakis et al., 2021b; Waller et al., 2022) will enable detailed exploration of various aspects of the symbiotic interaction in detail. Firstly, one could use this system to clarify how nitrogen starvation preconditions the host plants to attract and establish the initial interaction with the cyanobiont. It was hypothesized that symbiont attraction could have evolved by the extension of a conserved nutrient starvation response mechanism (Isidra-Arellano et al., 2021). Furthermore, forward genetic experiments could be used to identify the genes necessary for the initiation, establishment, and stabilization of the symbiotic interaction. Finally, reverse genetic tools could be employed to functionally verify the effect of candidate genes. Collectively, the hornwort-cyanobacteria system provides a tractable tool to thoroughly explore the origin and evolution of plant-cyanobacteria interactions.

It is expected that this knowledge can be used in the future to engineer crops capable of initiating the mutualistic interaction with cyanobacteria (Liu and Rousk, 2022; Rousk, 2022). This type of interaction has received increasing interest in recent years due to its significant translational potential to boost crop yield without applying additional artificial fertilizer (Alvarenga and Rousk, 2022; Rousk, 2022). Compared to AM fungi, cyanobacteria, especially the members of the genus *Nostoc* dominantly present in hornworts, are less dependent on the host, do not necessarily require specialized plant structures like arbuscules, and therefore hold a promising translational potential (Adams and Duggan, 2008).

### 5.6 Photoreceptors and flavonoids

Hornworts have a unique photoreceptor called neochrome, which is a chimeric gene composed of a red/far-red-sensing module from a phytochrome and a blue-sensing phototropin (Li et al., 2014; Li et al., 2015). Apart from hornworts, neochrome is only found in ferns (**Figure 3A**). Interestingly, it has been proposed that ferns acquired neochrome by horizontal gene transfer from hornworts (Li et al., 2014). It remains to be understood whether the function of neochrome in hornworts is similar to ferns, where it increases light sensitivity by perceiving both red and blue light signals and ultimately mediates phototropism and chloroplast relocation (Kawai et al., 2003; Suetsugu et al., 2005; Li et al., 2014; Li et al., 2015).

Hornworts do not produce flavonoids (Davies et al., 2020) (**Figure 3A**). Flavonoids are polyphenolic secondary metabolite compounds with approximately 8000 being reported so far in land plants (Andersen and Markham, 2005). Flavonoids play a wide variety of important roles, ranging from UV radiation and pathogen protection to providing color to flowers and fruits to attract pollinators and seed dispersers. It is hypothesized that the evolution of flavonoid biosynthesis pathways coincided with the transition of plants to terrestrial ecosystems and that flavonoid biosynthetic pathways were present in the common ancestor of all land plants. The presence of degenerated sequences of genes encoding enzymes that catalyze the initial steps of the flavonoid biosynthesis pathway in *A. agrestis* and *A. punctatus* genomes suggest that the absence of flavonoids in hornworts likely represents a secondary loss. However, functional analysis is needed to confirm whether or not those genes that are present in hornworts can compensate for homologous genes in other plant groups and still produce functional enzymes.

## 6 Current challenges and conclusions

While significant advancements have been made in the last ten years in the genetic manipulation of hornworts, many challenges remain to be tackled in the future. Testing and optimization of a CRISPR/Cas9-based genome editing method is underway but has not yet been reliably established. Genetic editing using transient expression of CRISPR/Cas9 components in protoplasts appears feasible, but progress is currently hindered by the low regeneration potential of protoplasts. Preliminary experiments suggest that genetic transformation *via* homologous recombination may be possible. Nevertheless, further experiments are needed to identify the optimal parameters required. Until now, no inducible promoter systems have been tested in the hornworts. Further progress should be made on the long-term storage of hornwort plants. While spores provide a potential agent for long-term storage, many hornwort species do not develop sporophytes under laboratory conditions. Therefore, conditions necessary for long term storage of gametophyte fragments must be established. It is also necessary to extend the available genomic and transcriptomic resources, and efforts in that direction are currently underway. Finally, functional annotation of the hornwort genes is still in its initial phase. Considerable efforts must be directed towards the functional characterization of genes. This could be partially achieved by applying forward genetic techniques involving mutagenesis. Classical genetic approaches using genetic mapping *via* crossing of genetically diverse individuals must be also established.

*A. agrestis* has already been adopted by several groups as an experimental system and appears in literature with increasing frequency. The development of additional tools, especially CRISPR/cas9 technology, will spark further interest in hornworts and will further facilitate research in comparative developmental studies across bryophytes and vascular plants to answer long-standing questions of plant evolution and plant biology in general.

## Author contributions

EF, AOM, MW, AN, SWT, YY, SR, LW, KS and PS collaboratively wrote the manuscript. EF, PS and MW coordinated the writing and finalized the manuscript. All authors contributed to the article and approved the submitted version.
